# A German national prevalence study on the cost of intensive care: an evaluation from 51 intensive care units

**DOI:** 10.1186/cc5952

**Published:** 2007-06-26

**Authors:** Onnen Moerer, Enno Plock, Uchenna Mgbor, Alexandra Schmid, Heinz Schneider, Manfred Bernd Wischnewsky, Hilmar Burchardi

**Affiliations:** 1 Department of Anaesthesiology, Emergency and Critical Care Medicine, University of Göttingen, Robert-Koch-Straße 40, Göttingen 37075, Germany; 2 HealthEcon Ltd, Steinentorstraße 19, Basel 4051, Switzerland; 3 Faculty of Mathematics and Computer Science, University of Bremen, Bibliothekstraße 1, Bremen 28359, Germany

## Abstract

**Introduction:**

Intensive care unit (ICU) costs account for up to 20% of a hospital's costs. We aimed to analyse the individual patient-related cost of intensive care at various hospital levels and for different groups of disease.

**Methods:**

Data from 51 ICUs all over Germany (15 primary care hospitals and 14 general care hospitals, 10 maximal care hospitals and 12 focused care hospitals) were collected in an observational, cross-sectional, one-day point prevalence study by two external study physicians (January–October 2003). All ICU patients (length of stay > 24 hours) treated on the study day were included. The reason for admission, severity of illness, surgical/diagnostic procedures, resource consumption, ICU/hospital length of stay, outcome and ICU staffing structure were documented.

**Results:**

Altogether 453 patients were included. ICU (hospital) mortality was 12.1% (15.7%). The reason for admission and the severity of illness differed between the hospital levels of care, with a higher amount of unscheduled surgical procedures and patients needing mechanical ventilation in maximal care hospital and focused care hospital facilities. The mean total costs per day were €791 ± 305 (primary care hospitals, €685 ± 234; general care hospitals, €672 ± 199; focused care hospitals, €816 ± 363; maximal care hospitals, €923 ± 306), with the highest cost in septic patients (€1,090 ± 422). Differences were associated with staffing, the amount of prescribed drugs/blood products and diagnostic procedures.

**Conclusion:**

The reason for admission, the severity of illness and the occurrence of severe sepsis are directly related to the level of ICU cost. A high fraction of costs result from staffing (up to 62%). Specialized and maximum care hospitals treat a higher proportion of the more severely ill and most expensive patients.

## Introduction

Intensive care units (ICUs) currently represent the largest clinical cost centres in hospitals, with expenses estimated to reach up to 20% of a hospital's budget [1]. The total cost per ICU patient highly depends on the severity of illness and the length of the ICU stay [2-5]. Complications and the need for prolonged mechanical ventilation lead to an increase in diagnostic procedures, invasive monitoring and the amount of drugs and blood products, and thus lead to an increase of the daily cost per patient [2,3,5-10]. The prolonged length of stay in this resource and the personnel-intensive environment results in overall costs that, for example, in septic patients are two-fold to 11-fold higher compared with the general cost per patient [5,9,11,12]. Costs for personnel make up 30–69% of the total cost per patient [11,13-20]. Besides the high impact of fixed personnel and overhead costs, direct variable costs are very important to consider in order to understand the cost of an ICU patient. Depending on the therapeutic and diagnostic needs of the patient, these direct variable costs can vary a lot [21-25]. Knowing the cost of what we prescribe [26,27] might lead to a more rational but not restricted intensive care [28] and is the basis for studies on cost-effectiveness [29,30].

Despite the need for cost data, there are still few investigations on the real costs of intensive care therapy based on the individual patient's resource usage [21,22,25,31,32]. Moreover, the comparison of studies on the cost of ICU care is often difficult due to the use of varying methods of cost calculation [30,33,34]. Many studies are based on ICU annual expenditures or budgets, from which the costs are broken down for the patient numbers and the days spent in the ICU [15,35-38], charges [6,39] or cost-to-charge ratios [6,10,40-42] to analyse the cost of ICU care. Furthermore, most of these studies were performed in university hospitals or maximal care facilities and only a few investigations include hospitals of a lower level of care [17,20,43,44]. To our knowledge there are no studies directly comparing the costs of intensive care from hospitals of different levels of care.

The present study, endorsed by the German Interdisciplinary Association of Critical Care Medicine (DIVI), was performed by visiting a nationwide randomly selected representative sample of ICUs from various hospital levels of care. The study is a one-day cross-sectional survey, collecting data on all patients with a length of stay > 24 hours who were treated in the ICU. Based on these data we aimed to obtain detailed information on the costs of intensive care in German ICUs from different levels of hospital care. Part of the data obtained was presented at the congress of the European Society of Intensive Care Medicine in 2004 [45].

## Methods

After written consent from the hospitals' administration and from the head of the ICUs to take part in our study and with local ethics committee approval, two independent interviewers visited 51 representatively selected ICUs in hospitals all over Germany between January and October 2003. Hospital selection was based on a nationwide prevalence study on sepsis performed by the German Competence Network Sepsis, for which a representative hospital sample was randomly selected from the registry of German hospitals and stratified by size [46]. From this larger study sample (454 ICUs in 310 hospitals out of 2,075 ICUs in 1,380 hospitals) a smaller sample of 51 ICUs, representing 2.5% of all ICUs in Germany, was randomly selected by the German Competence Network Sepsis administration. Four ICUs refused to take part in the study and thus were replaced by further randomly chosen ICUs according to their hospital size.

The ICUs included were defined by four levels of hospital care (Table 1). The allocation of hospital levels is based on the governmental mandate of medical care provision and mainly differs in terms of medical specialties and of diagnostic and therapeutic possibilities provided.

**Table 1:** Type of hospital, size and number of included patients (*n *= 453)

Hospital category (type of care)	Hospitals (*n*)	Hospital size (number of beds)	Number included (*n*)	Intensive care unit size (number of beds)	Intensive care units (*n*)	Included patients (*n*)
Primary care	15	0–200	8	0–5	2	93 (20.5%)
		201–500	7	6–10	12	
		501–1'000	0	11–15	1	
		>1'000	0	16–20	0	
				>20	0	
General care	14	0–200	4	0–5	1	103 (22.7%)
		201–500	10	6–10	11	
		501–1'000	0	11–15	2	
		>1'000	0	16–20	0	
				>20	0	
Maximum care	11	0–200	0	0–5	0	146 (32.3%)
		201–500	1	6–10	3	
		501–1'000	5	11–15	6	
		>1'000	5	16–20	1	
				>20	1	
Focus care	11	0–200	2	0–5	0	111 (24.5%)
		201–500	5	6–10	5	
		501–1'000	4	11–15	3	
		>1'000	0	16–20	3	
				>20	0	

Primary care hospitals (pcH) contribute to the primary healthcare in the very local area and provide the 'basic' specialties such as surgery and/or internal medicine; in addition, pcH often offer other specialties (for example, gynaecology and obstetrics).

General care hospitals (gcH) provide care for a broader area. Besides internal medicine and surgery, these hospitals may – based on governmental requirements planning – also house specialties such as gynaecology and obstetrics, ear, nose and throat, ophthalmology and, eventually, orthopaedics, urology, radiology and laboratory services.

Focus care hospitals (fcH) assure healthcare on a regional level. In contrast to gcH facilities, they may also include departments such as neurology, paediatrics, or psychiatry. According to the requirements they may provide very focused care (for example, heart or thoracic surgery).

Maximum care hospitals (mcH) provide highly differentiated diagnostic and therapeutic possibilities (for example, computer tomography, magnetic resonance tomography). University hospitals are generally maximum care hospitals.

Sorted by category, the present study included 14 surgical ICUs, five nonsurgical ICUs, 28 interdisciplinary ICUs and four special ICUs.

### Data collected

Structural ICU data (such as the number of ICU beds, ICU staffing) and hospital data (such as the number of hospital beds and hotel cost) were collected by interviewing the head of the ICU, the head of the hospital's administration and the assigned ICU physician.

We included all adult patients with a length of ICU stay ≥ 24 hours who were treated in the selected ICU on the day of the analysis. Clinical and resource use data were collected on a cross-sectional (one-day) basis by analysing the patient records and by interviewing the nurses and physicians involved in the treatment of the patient.

Resource data included the complete ICU therapy (every drug, invasive procedures, blood and blood products and fluid therapy), the usage of disposables (drainages, dressings, and so on) and diagnostic procedures such as X-ray scan, computed tomography scan, laboratory testing and microbiological analysis. The clinical patient data collected consisted of age, sex, reason for admission, diagnosis, comorbidities and type of patient (nonsurgical, scheduled surgery, unscheduled surgery). The severity of illness, measured by the Simplified Acute Physiology II (SAPS II) score, the System Organ Failure Assessment (SOFA) score and the existence of sepsis, as well as the workload (by the Therapeutic Intervention Scoring System (TISS)-28) were determined by the visiting physician for the day of the analysis. After the initial visits, the ICUs were later contacted by telephone to obtain follow-up information (total length of ICU stay and hospital stay, and ICU/hospital survival) of the selected patients. After this second contact, a list of the assessed resources was sent to the hospitals with a request to provide the hospital-specific (purchasing) prices and costs.

### Cost calculation

The cost perspective of this study was the selected ICU from the hospital's point of view combining two approaches to obtain the individual patient's specific costs.

#### Variable cost

For every patient, all resources used on the visiting day (excluding staff time) – that is, the type and frequency of given drugs and consumables (syringes, catheters, and so on) as well as laboratory and microbiological analyses and diagnostic procedures – were assessed on an individual basis. Procedures outside the ICU (for example, surgical interventions) on the day of analysis were not taken into account, except for X-ray scans, computed tomography scans, and so forth.

The direct variable costs were calculated based on a specific cost catalogue for the whole ICU sample using the following approach. Prior to the study, items needed for complex procedures – for example, the insertion of a venous access (venous catheter, gloves, swab, and so on) – were defined at the ICU in Göttingen. Actual hospital purchasing costs for drugs, disposables, nutrition, and blood products were collected in a hospital-specific cost catalogue. Per-package costs were transformed into unit costs when necessary. Due to the fact that not every one of the 51 hospitals was able to provide detailed information on every single item, a general cost catalogue was established and used for cost calculation. Effective costs of laboratory and microbiologic analysis were generally not available (only the much higher official tariffs). For these items, the detailed information of effective costs from the University hospital of Göttingen was used under the assumption that existing cost differences between institutions are negligible. Costs per patient resulted from multiplying the mean prices (from the cost catalogue) by the frequencies and dosages of resources used for the individual patients derived from the assessed data.

#### Fixed cost

Intensive care staff costs per day of care were calculated for each centre by multiplying the wages (based on gross income, employer's contribution included) per hour by the data on staff numbers, working hours, and ICU-related work percentages on weekdays and weekends of the individual ICUs, obtaining the mean local staff costs per day. Physicians for consultation were not included as long as they were not part of the ICU staff. Total fixed costs were allocated to the number of ICU beds.

Basic bed costs per day ('hotel costs') include overhead costs for nonclinical support services, maintenance, energy and hospital administration. This information was derived from the hospital administration. Equipment (such as monitors, ventilator, and so on) and other investment costs as well as depreciation were not included.

Total patient costs were obtained by adding up the calculated direct variable cost and the fixed cost. All costs were gathered and are presented in euros (for 2003).

### Data analysis

Analysis of data was carried out using MS Access 97 and Excel 7.0 (Microsoft Corporation, Redmond, WA, USA), SPSS 11.0 and Clementine (classification and regression algorithm trees: C4.5 and CART) (SPSS Inc., Chicago, IL, USA) [47]. Differences in costs of subgroups, length of stay (LOS), and mortality were tested statistically; for example, using chi-squared statistics to identify optimal splits or using CART methods, which are based on minimization of impurity measures (for example, the Gini index). The Gini index is a measure based on squared probabilities of membership for each target category in the node [48]. The index reaches its minimum (zero) when all cases in the node fall into a single target category. We applied Bonferroni adjustment to *P *values when multiple tests were performed. We used this adjustment to prevent the overall error rate from exceeding the nominal criterion (alpha) due to multiple tests. Cost data are presented as the mean with standard deviation, while clinical data are given as the median and 25th and 75th percentiles unless stated otherwise.

## Results

### Patient data

A total of 453 patients with a length of stay of ≥ 24 hours were included; 35.8% (*n *= 162) were nonsurgical patients, 32.2% (*n *= 146) were scheduled surgery patients, and 32% (*n *= 145) were patients with unscheduled surgery (Table 2). On the day of assessment, 13.7% (*n *= 62) of the patients were found to be severely septic, 41.7% were mechanically ventilated, and 4.2% received renal replacement therapy (Table 2). The overall ICU mortality was 12.1% (*n *= 55). ICU mortality tended to be higher in pcH patients (18.3%), but did not reach significance. The type of admission differed (*P *< 0.0001) between hospital levels, with the highest percentage of scheduled surgical patients being treated in fcH (49.6%) (Table 2). The rate of unscheduled surgical procedures was highest in mcH (37.7%) followed by gcH (34%). The pcH had the highest share of nonsurgical patients (59.1%).

**Table 2:** Patient data sorted by level of hospital care

	All patients	Primary care hospitals	General care hospitals	Focused care hospitals	Maximum care hospitals
Patients (% (*n*))	100 (453)	20.5 (93)	22.7 (103)	24.5 (111)	32.2 (146)
Age (years) (median (Q1–Q3))	68 (56.8–76.0)	69.0 (60.0–78.0)	71.0 (58.0–78.5)	68.0 (60.0–75.5)	68.0 (53.3–73.0)
					
Gender (% (*n*))					
Male	55.2 (250)	46.2 (43)	46.6 (48)	63.1 (70)	61.0 (89)
Female	44.8 (203)	53.8 (50)	53.4 (55)	36.9 (41)	39.0 (57)
Admission from (% (*n*))					
Operation room	36.0 (163)	26.9 (25)	39.8 (41)	58.6 (65)	63.0 (92)
General ward	14.1 (64)	33.3 (31)	21.4 (22)	15.3 (17)	16.4 (24)
Emergency ward/ambulance	21.0 (95)	35.5 (33)	32.0 (33)	5.4 (6)	15.8 (23)
Other hospital/intensive care unit	8.4 (38)	4.3 (4)	3.9 (4)	20.7 (23)	4.8 (7)
Readmission	6,8 (31)	4.3 (4)	6.8 (7)	7.2 (8)	8.2 (12)
Reason for admission (% (*n*))					
Nonsurgical	35.8 (162)	59.1 (55)	42.7 (44)	24.3 (27)	25.3 (37)
Surgical scheduled	32.2 (146)	12.9 (12)	23.3 (24)	49.6 (55)	37 (54)
Unscheduled surgery	32.0 (145)	28.0 (26)	34.0 (35)	26.1 (29)	37.7 (55)
Severity of illness (median (Q1–Q3))					
SOFA score	4 (2–6)	3 (2–6)	3 (2–6)	3 (2.5–7)	4 (2–7)
SAPS II score	32 (23–44)	35 (23–46)	30 (20–40.5)	30 (21.5–44.5)	31 (25–45.8)
					
Workload, TISS score (median (Q1–Q3))	27 (19–35)	23 (16–30)	24 (18–29)	27 (19–36)	33 (24–38)
High resource intensive (% (*n*))					
Mechanical ventilation	41.7 (189)	30.1 (28)	24.3 (25)	47.8 (53)	56.9 (83)
Renal replacement therapy	4.2 (19)	1.1 (1)	1.9 (2)	5.4 (6)	6.9 (10)
Invasive procedures^a^	52.5 (238)	46.2 (43)	43.7 (45)	56.8 (63)	59.6 (87)
Antibiotic/mycotic treatment	55.9 (253)	51.6 (48)	49.5 (51)	56.8 (63)	62.3 (91)
Blood products	13.7 (62)	10.8 (10)	10.7 (11)	17.1(19)	15.1 (22)
Diagnostic procedures^b^	93.8 (425)	93.6 (87)	94.2 (97)	91 (101)	95.2 (139)
Length of stay (days) (median (Q1–Q3))					
Intensive care unit	6 (2–18)	5 (2–11)	4 (1–9.5)	12 (3–26)	6 (2–19)
Hospital	22 (14–40)	19 (11–33)	20 (13.5–34)	29 (17–53)	23 (14–36.8)
					
Mortality (% (*n*))					
Intensive care unit	12.1 (55)	18.3 (17)	6.8 (7)	10.8 (12)	13 (19)
Hospital	15.7 (71)	21.5 (20)	8.7 (9)	16.2 (18)	16.4 (24)

Data are presented as median values (25th (Q1) and 75th (Q3) percentiles) or percentages. SAPS, Score Simplified Acute Physiology, SOFA, System Organ Failure Assessment; TISS Therapeutic Intervention Scoring System (TISS-28).

^a^For example, intubation, catheterizations, renal replacement therapy and mechanical ventilation.

^b^All diagnostic procedures including imaging, laboratory tests and microbiologic analysis.

The workload measured by TISS-28 was significantly higher in mcH (median 33, 24 to 38) and fcH (median 27, 19 to 36) compared with pcH (median 24, 16 to 30) and gcH (median 23, 18 to 29) (*P *< 0.0001) (Table 2). There were also significant differences in frequencies of mechanical ventilation between the hospital levels of care (*P *< 0.0001): 56.9% in mcH, 47.8% in fcH, 24.3% in gcH, and 30.1% in pcH.

The ICU LOS and the hospital LOS differed significantly (*P *= 0.001) and was highest in fcH (median ICU LOS, 12 days; hospital LOS, 29 days), while mcH (median ICU LOS, 6 days; hospital LOS, 23 days), gcH (median ICU LOS, 4 days; hospital LOS, 20 days), and pcH (median ICU LOS, 5 days; hospital LOS, 19 days) only showed slight differences.

Severity of illness on the day of analysis, measured by SAPS II and SOFA scores, was 32.0 (23 to 44) and 4.0 (2 to 6), respectively. The SAPS II score and the SOFA score did not differ substantially between hospital levels (Table 2).

There was a difference in the percentage of patients admitted from other hospitals/ICUs, with the highest rate of transferred patients in fcH (20.7%) (Table 2) but with only slightly higher admission rates in mcH compared with gcH and pcH. Between the different levels of care, the characteristics of transfer patients differed significantly as shown by the severity of illness SAPS II score (pcH, 39 (34 to 44); gcH, 33 (25 to 39); fcH, 29 (27 to 43); mcH, 47 (43 to 58); *P *= 0.0328).

### ICU structure

The staffing structure of nurses did not differ between the different levels of hospital care (*P *= 0.732) with regard to the nurse-to-bed ratio per shift (median, 0.37 (0.33 to 0.43); mean, 0.39 ± 0.1). Staffing structure of physicians, however, differed between the various levels of care. In the mcH ICUs 91% of ICU physicians spent 80 to 100% of their working time in the ICU, with no other responsibilities for 73% of the physicians. At the other hospital levels, the percentage of full-time ICU physicians (80 to 100% of working time) was lower: 30% in pcH, 55% in gcH, and 55% in fcH.

### Cost calculations

The mean total costs per patient and day were €791 ± 305. Staff costs comprise the largest proportion of total costs at around 56%, followed by medication costs (including blood products, fluids, nutrition, drugs) at 18.7% (Table 3). The mean cost per TISS point was €32 ± 13.7. The mean daily cost in various subgroups of patients differed considerably. Patients admitted for unscheduled surgery were more expensive (€829 ± 318) than scheduled surgery patients (€785 ± 320) or nonsurgical patients (€759 ± 277) (*P *= 0.004). Patients on mechanical ventilation caused higher costs than nonventilated patients (€946 ± 355 versus €680 ± 203; *P *< 0.0001). Septic patients had consistently higher daily costs than nonseptic patients in all hospital levels of care, with an average of €1,090 ± 422 versus €745 ± 255 (*P *< 0.0001).

**Table 3:** Mean daily intensive care unit costs per patient and percentage of total cost sorted by hospital level of care

Direct cost (€)	All hospitals (*n *= 453)	Primary care hospitals (*n *= 93)	General care hospitals (*n *= 103)	Focused care hospitals (*n *= 111)	Maximum care hospitals (*n *= 146)
Mean daily cost	791 (305)*	685 (234)	672 (199)	816 (363)	923 (306)
€/TISS point	32 (13.7)	33 (14.8)	31 (11.6)	33 (15.0)	32(13.7)
Staff cost	444 (105)*	387 (54)	415 (93)	438 (114)	505 (101)
Medication^a^	148 (191)*	139 (185)	98 (108)	169 (211)	174 (217)
Antibiotics	24 (43)	20 (36)	19 (38)	29 (48)	25 (46)
Blood products	30 (105)	16 (60)	16 (53)	43 (151)	39 (113)
Invasive procedures^b^	24 (55)	14 (30)	20 (50)	30 (66)	30 (61)
Diagnostics^c^	96 (90)*	76 (66)	67 (64)	102 (97)	122 (105)
Laboratory	65 (62)*	49 (40)	44 (37)	71 (67)	87 (75)
Microbiology	9 (30)	7 (25)	3 (8)	9 (27)	16 (41)

Data presented as the mean (standard deviation). TISS, Therapeutic Intervention Scoring System (TISS-28).

^a^All given drugs, fluids, blood products and nutrition.

^b^For example, intubation, catheterizations, renal replacement therapy and mechanical ventilation.

^c^All diagnostic procedures including imaging, laboratory tests and microbiologic analysis.

*Significant difference (*P *< 0.05) between the different levels of hospital care.

We found a clear group separation of costs in patients with SAPS II score < 47 (*n *= 363, €742 ± 252) versus SAPS II score ≥ 47 (*n *= 90, €984 ± 410) (*P *< 0.0001). Organ failure assessed by the SOFA score also showed a clear separation of costs at SOFA score < 7 (*n *= 369, €728 ± 240) versus SOFA score ≥ 7 (*n *= 84, €1,061 ± 402) (*P *< 0.0001).

Survivors were less expensive than nonsurvivors (€773 ± 291 versus 914 ± 369 per day; *P *= 0.012).

In 45 patients (10% of patients) representing the highest cost group (upper 90th percentile), the mean daily cost was €1,470 ± 308. The spectrum of these patients was mainly represented by cases with unscheduled surgery (40%) that were mostly mechanically ventilated (86.7%) and suffered from sepsis (44.4%).

### Comparison of costs between hospital levels of care

In general, mcH and fcH had significantly (*P *< 0.0001) higher mean patient costs per day than smaller hospitals with primary and general care (Table 3). Patients with long ICU LOS (>14 days) caused a significantly (*P *< 0.0001) higher daily cost (€917 ± 392) compared with those with shorter ICU LOS (€735 ± 241). In the group of long ICU LOS patients, the mean daily cost also varied significantly between the different levels of hospital care: €776 ± 210 in pcH, €793 ± 308 in gcH, €865 ± 449 in fcH, and €1,089 ± 370 in mcH (*P *= 0.0019). Namely, 84.4% of the most expensive patients (upper 90th percentile) were treated in mcH and fcH. The higher expenditures are reflected by the difference in workload (TISS) (Table 2), which was significantly higher in mcH (mean, 31.6) and fcH (mean, 27.3) (*P *< 0.0001). However, the calculation of the cost per TISS point revealed no significant differences (*P *= 1.000).

Within the small fraction of patients transferred from other ICUs or hospitals (Table 2), those transferred to mcH caused the highest daily cost (€1,051 ± 262) compared with the other levels of care (pcH, €714 ± 299; gcH, €683 ± 144; fcH, €621 ± 234) (*P *= 0.0021). The staff costs differed significantly between the hospital levels of care (*P *< 0.0001) and were the highest in mcH (Table 3). Significant differences were also found in the expenditures for diagnostic procedures (*P *< 0.0001), laboratory investigations (*P *< 0.0001), microbiology (*P *= 0.0062), and for medication in general (*P *= 0.0088). The costs for blood products (*P *= 0.2054), invasive procedures (*P *= 0.0785), and antibiotics (*P *= 0.3205) were similar (Table 3).

Nonsurgical and special focus ICUs showed higher total daily costs compared with surgical and interdisciplinary ICUs.

## Discussion

The present study aimed to estimate the current situation in German ICUs over all levels of care. This was achieved by visiting 51 representatively selected ICUs across Germany, covering all types of care (general, basic, maximum, main focus).

Mean total intensive care expenditure per patient per day in Germany was €791 ± 305, with 19.4% of the patients costing more than €1,000 per day and a maximum of €2,815 per patient-day. Studies from previous studies from different European countries found mean daily costs ranging between €1.125 and €1.590 per day [14,16,31,32,35,36,49]. The majority of these studies were performed in university or teaching hospitals or did not break down the cost in order to compare different levels of hospital care [17,22,35,43,49]. Taking only university hospitals into account we found a mean cost of €1.132, which is well in line with the abovementioned recent findings. The overall lower mean daily cost of €791 compared with these studies can therefore be easily explained by the high number of hospitals of levels other than maximum care.

As shown in a number of studies, the severity of illness has a huge impact on ICU cost [2,3,5-10,50]. In our study population, 10% of all patients (45 patients; mean cost, €1,469) consumed about 19% of the total resources. In all levels of care the most expensive patients were those needing mechanical ventilation, those patients having a high severity of illness and/or severe sepsis as well as nonsurvivors. Patients admitted for unscheduled surgical procedures (that is, emergency cases) caused significantly increased cost.

In previous studies the following daily costs were found for septic patients: €1,318 (in 2001 from three German teaching hospitals) [5], and US$931 (in 1998 from one teaching hospital in the United Kingdom) [11].

Overall there was no difference in SAPS and SOFA scores on the study day between the hospital levels. We have to bear in mind, however, that these scores were evaluated during intensive care treatment. The lack of difference therefore only indicates a more or less stable situation during the treatment in general, not the primary severity of illness. Nonetheless, the patients treated in mcH were obviously more severely ill than those in smaller hospitals: cases needing mechanical ventilation were nearly twice as frequent in mcH as in pcH, and renal replacement therapies and other invasive procedures were more frequent in mcH. Emergency cases with unscheduled surgery requiring more intensive care interventions were also more frequent in mcH. Consequently, the TISS was higher in mcH and 84.4% of the most expensive patients (that is, the upper 90th percentile of costs) were treated there. Prediction of patients' average daily costs in intensive care, however, is only scarcely linked to descriptive criteria. Only 33.6% of the variation of daily costs (mean ± SD, €704 ± 422) in a monocentre analysis could be explained by criteria such as the Acute Physiology and Chronic Health Evaluation II score, gender, age, mechanical ventilation, emergency admission and others [21].

Resource consumption and the use of diagnostic procedures differed significantly between the hospital levels. Related to the level of performance measured by the TISS-28, however, the overall mean daily ICU cost per patient was €32.0/TISS point with only minor differences between the hospital levels (Table 3). This shows that mcH are not at all more expensive if matched against the level of performance. This profile of daily cost per TISS point is slightly less than the values of €34–37/TISS recently evaluated from a single university ICU in Finland [38] or of €36/TISS in Germany [16].

The differences between hospital categories are explained by the allocation of various hospital responsibilities. This is reflected by the patients transferred between hospitals of different levels of care. Patients transferred to maximum care ICUs were more severely ill and more expensive compared with those transferred to pcH or gcH. This partly confirms the recent findings of Golestanian and coworkers, who showed that patients transferred to tertiary care centre ICUs were more severely ill and more expensive [51]. This reflects a common practice that patients who cannot be handled in primary or general care facilities due to limited diagnostic or therapeutic capabilities are usually transferred to fcH or mcH ICUs for more effective treatment [52-54]. On the other hand, patients who are successfully treated in fcH or mcH are often transferred back for further intensive care treatment in the lower level hospitals, often due to the lack of local intermediate care facilities.

The cost for staffing is the highest expenditure of intensive care treatment, with 56.1% on average overall (Table 3). Staffing of nurses is remarkably similar at all hospital levels. This is a consequence of official regulations on staffing for nurses, which is related to the number of intensive care beds. There are, however, no such strict regulations for physicians. In general, the larger ICUs in mcH are mostly run by full-time physicians, whereas in smaller hospitals the ICU allocation of physicians to the ICU is more reduced and they often have additional tasks (for example, in the operating theatre). Consequently, the mcH are burdened with the highest staffing cost.

To our knowledge this is the first study that compared the ICU cost nationwide in intensive care in a representative sample of 51 ICUs by analysing the resource consumption on an individual patient level. It must be mentioned that this bottom-up approach is very laborious and probably difficult to perform in studies analysing cost in a larger number of ICUs over the ICU stay. Alternatives such as cost blocks proposed Edbrooke and colleges [17,24,33,55], cost analysis based on the therapeutic score [16,44,56-58] or cost prediction models [59] might be more applicable in daily practice. These methods should only be considered after carefully testing for accuracy on a national level, however, and are less reliable on the individual patient basis [13]. With the increasing number of computerized patient data management systems in the ICU, the analysis of direct variable cost becomes easier [60]. Besides the relatively large number of ICUs included in our study, there are further strengths one could consider. The ICUs were included based on a stratified random sampling strategy and the data were collected by two dedicated intensivists visiting the ICUs instead of sending out data sheets to collect probably inhomogeneous information.

There are also certain limits caused by the study design. The study was performed as a 1-day prevalence investigation that may provide accurate actual information. Owing to this design, however, we cannot draw conclusions on the total cost per patient. Moreover, the quality of care provided or its effectiveness cannot be estimated since important information on the course of ICU therapy is lacking.

In our study we only included patients with an ICU LOS longer than 24 hours. In Germany, an ICU's task within the hospital differs highly between the different levels of care. In smaller hospitals there are no intermediate care wards; therefore, postoperative recovery supervision and care in pcH and gcH is routinely provided by the ICUs. The higher level of personnel costs in maximum care facilities is mainly caused by the fact that critically ill patients are treated round the clock in maximum care ICUs.

ICUs are not only responsible for critically ill patients, however – especially in gcH and pcH – but also take care of so-called intermediate care patients. After regular working hours the ICU staff takes care of postoperative recovery patients. To avoid the inclusion of non-ICU patients, therefore, a LOS > 24 hours was defined as an inclusion criterion. We might therefore have missed extremely severely ill patients who died within the first hours after admission.

Recent studies have shown that increasing the ICU size [43] but also increasing the adequate ICU staffing can be considered cost-effective [61-63]. From our study it cannot be deduced that the higher resource usage and higher fixed cost in mcH may also be comparatively cost-effective in terms of outcome improvement. Such presumptions should be avoided because of differences in case mix between the hospital levels of care and because of the one-day prevalence study design. For this purpose, a matched-pairs study with comparable patient groups analysed over the whole period of the ICU stay is required.

For cost calculation, a cost catalogue based on averaged resource information from the participating ICUs was used. Owing to the confidentiality of such data, however, it was impossible to collect complete specific cost information on every item from each ICU. An averaged cost catalogue such as we used, then, might underestimate some differences in daily cost in such situations. For example, the purchasing price for a venous canula may vary by about 40% between different ICUs due to different brand and price conditions. Nevertheless, we suppose that the overall average cost catalogue may provide a sufficient basis for general cost calculations.

## Conclusion

The present study demonstrates that a considerable degree of variation exists between ICUs according to the hospitals' level of care. These differences are mainly caused by the case mix and by the need to provide a higher level of resource consumption for the cost of diagnostic procedures and of staffing in mcH. There are common cost patterns for certain patient groups independent of ICU or hospital categories, such as those with unscheduled surgical procedures. The need for prolonged mechanical ventilation as well as the occurrence of sepsis results in significantly increased cost per day.

## Key messages

• A high fraction of costs result from staffing (56.1% on average overall).

• Reason for admission, severity of illness and the occurrence of severe sepsis are directly related to the level of ICU cost.

• The case mix and workload (reflected by TISS score) significantly differs between different levels of hospital care.

• Specialized hospitals and mcH treat a higher proportion of the most expensive patients.

## Abbreviations

fcH = focused care hospitals; gcH = general care hospitals; ICU = intensive care unit; LOS = length of stay; mcH = maximum care hospitals; pcH = primary care hospitals; SAPS = Simplified Acute Physiology Score; SOFA = System Organ Failure Assessment; TISS = Therapeutic Intervention Scoring System.

## Competing interests

The study was supported by the German Interdisciplinary Association of Critical Care Medicine (DIVI), Lilly Deutschland GmbH, and departmental funds. Neither the German Interdisciplinary Association of Critical Care Medicine (DIVI) nor Lilly Deutschland GmbH has been involved in any part of the study or preparation of the manuscript. The authors declare that they have no competing interests.

## Authors' contributions

OM participated in conceiving and designing the study, carried out the hospital visits, data collection and data analysis, and drafted the manuscript. EP carried out the hospital visits and data collection. UM participated in the hospital visits and data collection. AS participated in data analysis and programming of the database. HS participated in the design of the study and data analysis. MBW performed the statistical analysis, and participated drafting the manuscript. HB conceived the study, participated in its design and coordination, and helped to draft and revise the manuscript.
